# COVID-19 Vaccine Hesitancy and Uptake among Nursing Staff during an Active Vaccine Rollout

**DOI:** 10.3390/vaccines9080858

**Published:** 2021-08-04

**Authors:** Lynn M. Baniak, Faith S. Luyster, Claire A. Raible, Ellesha E. McCray, Patrick J. Strollo

**Affiliations:** 1 Veteran Affairs Pittsburgh Healthcare System, Pittsburgh, PA 15240, USA; fsl3@pitt.edu (F.S.L.); Claire.Raible@va.gov (C.A.R.); Ellesha.McCray@va.gov (E.E.M.); strollopj@upmc.edu (P.J.S.); 2 Department of Health and Community Systems, School of Nursing, University of Pittsburgh, Pittsburgh, PA 15261, USA; 3 Division of Pulmonary, Allergy, and Critical Care Medicine, School of Medicine, University of Pittsburgh, Pittsburgh, PA 15261, USA

**Keywords:** Covid-19, vaccine uptake, nurses, United States

## Abstract

Even with the availability of COVID-19 vaccines, factors associated with vaccine hesitancy and uptake among nurses are unknown. This study evaluated COVID-19 vaccine hesitancy and uptake of nursing staff during one of the first COVID-19 vaccine rollouts in the United States. A cross-sectional survey was conducted during February 2021 among nursing staff working in a large medical center in central United States. There were 276 respondents; 81.9% of participants were willing to receive the vaccine during the initial rollout, 11.2% were hesitant, and only 5.1% were unwilling. The hesitant group was likely to report having inadequate information to make an informed decision about whether to receive the vaccine (45.2%) and about vaccine expectations (32.3%). The majority (83.3%) received at least one dose of the vaccine. Having greater than 10 years’ work experience (OR 3.0, 95% CI 1.16–7.9) and confidence in vaccine safety (OR 7.78, 95% CI 4.49–13.5) were significantly associated with vaccine uptake. While our study indicates higher vaccine uptake among nursing staff during an active vaccine rollout, there remains sustained hesitancy and unwillingness to uptake. For those hesitant to receive the COVID-19 vaccine, public health efforts to provide more data on side effects and efficacy may help increase vaccine uptake.

## 1. Introduction

COVID-19 is a significant public health crisis, with over 29 million cases and half a million deaths in the United States [1]. Mitigation strategies including social distancing, wearing a face covering, travel bans, complete or partial lockdowns, and other non-pharmacological interventions have been implemented to slow the rapid spread of COVID-19. Despite these effective strategies, recurrence of additional waves of infection have occurred. Efficacious vaccines are now available and the goal of herd immunity through vaccination along with exposure is at the forefront of achieving a reduction in hospitalizations and deaths and enabling a return to normalcy.

Vaccine hesitancy within the United States is a significant challenge [2,3]. Healthcare workers have been prioritized among the high-risk groups eligible for early vaccination. Healthcare workers play a pivotal role in providing care to infected patients and may be required to perform high-risk procedures increasing their risk of contracting the virus and potentiating the spread. Infection among healthcare workers results in a reduction in this essential workforce. In addition, healthcare workers serve as a role model of behavior and a trustworthy resource for vaccine-related information for patients [4]. Prior studies in healthcare workers have found concerning levels of uncertainty (up to 56%) and refusal (up to 25%) to receive a COVID-19 vaccine once it became available [5,6,7,8]. With the current availability of several COVID-19 vaccines, determining willingness and uptake of the vaccine along with concerns and determinants of sustained vaccine hesitancy among healthcare workers is important for developing effective and tailored communication strategies to ensure adequate vaccine coverage.

In this study, we examined the proportion of nursing staff working in a large medical center who were willing to receive a COVID-19 vaccine during an active vaccine rollout and their vaccine uptake rate. We also assessed attitudes and concerns and their relationships with willingness and uptake.

## 2. Materials and Methods

### 2.1. Study Design

A cross-sectional survey was conducted by administering an anonymous online questionnaire to nursing staff who work in a large healthcare facility in western PA during the month of February 2021. Study data were collected and managed using REDCap (Research Electronic Data Capture) [9,10]. REDCap is a secure, web-based software platform designed to support data capture for research studies, providing (1) an intuitive interface for validated data capture; (2) audit trails for tracking data manipulation and export procedures; (3) automated export procedures for seamless data downloads to common statistical packages; and (4) procedures for data integration and interoperability with external sources.

### 2.2. Sample Selection

Nursing staff belonging to specific work-related email groups received an initial e-mail invitation to participate in the study, followed by 2 email reminders throughout the course of the study. All emails contained a description of the study with the survey link. When each subsequent email reminder was sent out, the statement, “If you have already participated in the study, please disregard this email reminder” was added as a strategy to prevent respondents from participating more than once.

### 2.3. Variable and Data Collection

A literature review was conducted to inform development of the questionnaire. The survey consisted of a maximum of 26 questions. Response options included a combination of multiple choice, Likert scale, numerical, dichotomous, and open-ended free text. Contingency questions were included to obtain additional information from participants who already received the vaccine as well to those unwilling to receive the vaccine in order to explore vaccine uptake and hesitancy, respectively. The questionnaire addressed (1) sociodemographic data (age, sex, race/ethnicity, marital status, education, employment status, work location, work experience, and type of nursing staff), (2) vaccine hesitancy, (3) vaccine uptake, (4) concerns about the vaccine, (5) knowledge/attitudes about the vaccine, and (6) self-perceived risk of COVID-19. We assessed vaccine hesitancy for the COVID-19 vaccine with the question, “Are you willing to receive the COVID-19 vaccination?”, followed by the response options “yes, during the initial rollout” (i.e., willing), “yes, but choose to delay timing of injection” (i.e., hesitant), and “no” (i.e., unwilling). Participants who responded “no”, were asked the following open-ended question, “What makes you unwilling to receive the COVID-19 vaccination?” For those who were willing to receive the vaccine, we asked if they had already received the vaccine (yes/no) and those who replied ‘yes’ were asked to report any side effects they experienced. Work experience was categorized as ≤10 years or >10 years and confidence in safety of the vaccine was categorized as completely/fairly confident and somewhat/slightly/not at all confident. Expectations of the vaccine effectiveness was categorized as lifetime/limited immunity vs. reduction in symptom severity only or not at all effective. The final survey question was open-ended and asked if there was any additional information that the participant would like to share regarding thoughts or concerns about the COVID-19 vaccine.

### 2.4. Ethical Aspects

The study was conducted according to the guidelines of the Declaration of Helsinki and approved by the Institutional Review Board at the VA Pittsburgh Healthcare System (protocol code Pro00003710 and 15 January 2021). The study was deemed Exempt by the Institutional Review Board, which does not require a formal informed consent process. All surveys were anonymous, and participation was voluntary. Each participant received a written permission statement in the initial email invitation prior to taking the survey. All data provided to the investigators were fully deidentified.

### 2.5. Data Analysis

The sample distribution of demographic and vaccine-related variables was examined using descriptive analyses. Continuous variables were reported as mean ± standard deviation; categorical variables were described as numbers and percentages. Comparisons between those who received the vaccine and those who did not receive the vaccine were done using Student’s independent *t* test or Mann–Whitney U tests for continuous variables and chi-square tests (with the Fisher’s correction if less than five cases were present in a cell) for categorical variables. Binary logistic regression evaluated associations with receiving the COVID-19 vaccine (i.e., vaccine uptake; yes/no) among nursing staff. The model was adjusted for covariates identified from univariate analyses with a *p*-value < 0.05. Only cases with complete data were used. Results are reported as odds ratio (OR) and 95% confidence interval (CI). A *p*-value of < 0.05 was considered statistically significant. Analyses were performed using SPSS 26 Windows (IBM Corp., Armonk, NY, USA). Responses from open ended questions were reviewed and used to supplement the quantitative survey data. Open ended questions were intentionally created to be vague to enable more expansive responses. Qualitative responses were examined by C.R. using an Excel Spreadsheet to observe and group similar responses by tone (positive, negative, questioning/concerned) and general themes. Themes were then determined through compiling patterns of comparable responses. Subsequent themes were presented to the research team with concurrence by all members.

## 3. Results

### 3.1. Description of Sample

The survey was sent to 1300 nursing staff members and 276 (21.2%) completed the survey. Table 1 provides sample characteristics. The majority of participants were registered nurses (RN; 68.5%), female (83.7%), married (64.5%), White (81.5%), full-time (94.9%), and had greater than 10 years healthcare work experience (79.0%), with a mean average age of 48.3 (SD 10.2; range 23–69). Regarding educational degree, 60.2% had either an RN or licensed practical nurse degree while 33% had master’s degrees, 18.5% had an advanced practice nursing degrees, 5.4% had nursing assistant certificates, 3.6% had a doctorate of nursing practice, and less than 1% had a PhD.

**Figure 1:**
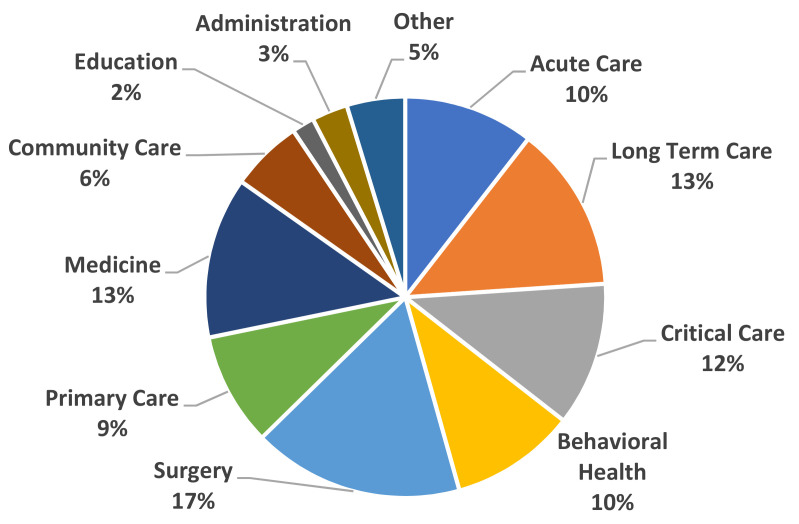
Survey participants by work unit.

Most nursing departments within the facility were well represented in the data (Figure 1).

During the administration of this survey, an active vaccine rollout was in progress at the healthcare facility. Overall, 81.9% of participants (*n* = 226) reported that they were willing to receive the vaccine during the initial rollout, 11.2% (*n* = 31) were hesitant, and only 5.1% (*n* = 14) were unwilling to receive the vaccine (Table 1). When assessing self-perceived risk of COVID-19 (Table 2), there was a higher than expected number of those who already had the disease (38.4%). Of those who did not report having COVID-19, most perceived their risk of contracting the disease as low, with 33.3% predicting that they would not get ill and 20.3% predicting a mild case (Table 2). Only 6.9% predicted that they would get seriously ill.

Overall, staff reported they were fairly or completely confident that the vaccine was safe (*n* = 204, 73.9%) and that it would effectively mitigate their risk (*n* = 199, 72.1%). The majority (*n* = 249, 90.2%) felt that they had adequate information to make an informed decision about whether to receive the vaccine. Primary sources of information that participants used to gather information on the COVID-19 vaccine were reported in order of frequency as: (1) professional organizations/journals, (2) government agencies, (3) professional peers, (4) employer, (5) mainstream media, (6) friends/family/informal networks, and (7) social media. The top three concerns of the vaccine among all participants were related to its side effects, the lack of evidence on its effectiveness, and the potential for the vaccine to be ineffective in mitigating risk.

### 3.2. Vaccine-Related Outcomes by Vaccine Hesitancy

Work experience of greater than 10 years was associated with an increased willingness to receive the vaccine. The hesitant and unwilling groups had a higher proportion of participants with less work experience (≤10 years) than those in the willing group (38.7% and 28.6% versus 18.6%, respectively; *p* = 0.03) (Table 1). No other significant differences were noted between groups regarding age, sex, race, marital status, or employment status.

In regards to vaccine-related outcomes (Table 2), both the willing and unwilling groups, compared to the hesitant group, were more likely to report having adequate information to make an informed decision about whether or not to receive the vaccine (96.0% and 100% versus 54.8%, respectively) and reported having adequate information about the expectations of the vaccine (96.8% and 92.9% versus 67.7%). Compared to the unwilling and hesitant groups, the willing group had a higher proportion of participants who reported that they were fairly or completely confident that the vaccine is safe (0% and 23.3% versus 85.7% respectively) and that it would effectively mitigate their risk (7.7%, and 35.5% versus 81.0% respectively; *p*-values ≤ 0.001). Willingness was significantly associated with the expectation that the vaccine would provide lifetime or limited immunity (*p* < 0.001). Those who were unwilling or hesitant were more likely to expect that the vaccine would only provide a reduction in symptom severity or be completely ineffective compared to those who were willing (69.2% and 48.4% versus 22.2%, respectively). Self-perceived risk of COVID-19 did not differ between groups (*p* = 0.14).

Of the participants who were hesitant to receive the vaccine, about half (*n* = 12) responded to the open-ended final survey question, “Is there any additional information you wish to share about your thoughts or concerns regarding the COVID-19 vaccine?” The major trends associated with the qualitative responses include effectiveness of the vaccine (33%), medical concerns (25%), and general fear of the unknown (25%). The following comment from one hesitant participant summarizes these trends,

“*My primary concern was that I felt the vaccine development was rushed and the emergency use agreement approval made me a little hesitant, not to mention it was a new vaccine and long-term side effects could not be researched as of yet. My other concern was other pre-existing health conditions I have and how the vaccine may impact those*.”

Other hesitant participants took an altruistic approach when answering the final question. One participant offered the following perspective,

“… *as a frontline healthcare worker who will soon be working in the Covid ICU, am I justified in taking that dose for myself in order to stay healthy, and am I better protecting my patients by being vaccinated or by saving a dose for those patients that are the most vulnerable?*……”

Some hesitant participants responded with more personal anecdotes, citing their own battle with the virus while also looking further into the future with what could happen with wide vaccine uptake,

“*As the vaccine does not prevent a person from becoming infected with COVID-19, there is little rush for me to get the vaccine. I have had COVID-19 which essentially is the same as getting the vaccine. Additionally, I feel that those who do receive the vaccine will effectively stop all precautions because they feel that they are completely protected from getting or giving the virus to someone else…… In light of the recent development of the new variations of the virus, I again feel that this vaccine will do nothing but potentially increase the number of cases*.”

### 3.3. Associations with Vaccine Uptake

Upon survey completion, 83.3% (*n* = 230) of participants had received at least one dose of the vaccine (Table 3). Of those who were hesitant (*n* = 31) to receive the vaccine during the initial rollout, 7 (22.6%) had received at least one dose of the vaccine at the time of survey completion. Of the 226 who were willing to receive the vaccine during the initial rollout, 222 (98.7%) had received at least one dose of the vaccine. Out of the seven who were hesitant but received the vaccine, six stated they were a great deal, or somewhat knowledgeable about the vaccine development process and that they had adequate information about the expectations of the vaccine. All seven were registered nurses who stated that their primary source of information regarding COVID-19 was from professional organizations.

The final regression model contained six total significant variables identified from Chi-square tests: (1) adequate information about the expectations of the vaccine, (2) adequate information to make an informed decision about whether to receive the vaccine or not, (3) confidence in safety, (4) confidence in effectiveness, (5) expectations of effectiveness, and (6) work experience (Table 3).

Although knowledge about the development process was significantly associated with vaccine uptake, we chose not to include this variable as a covariate because of its overlapping content (Table 4). The full model was statistically significant, χ2 (6, *n*  =  259)  =  78.3, *p*  <0.001 and explained 46.6% (Nagelkerke R square) of the variance in vaccine uptake. Having greater than 10 years of work experience in healthcare (OR 3.0, 95% CI 1.16–7.9) and having more confidence in the safety of the vaccine (OR 7.78, 95% CI 4.49–13.5) remained significantly associated with an increased likelihood of having received the vaccine.

Those who were willing and had received the vaccine, responded more positively to the final survey question asking about concerns or thoughts related to the vaccine. Among the positive tone, the following trends were established: active recommendation to others to receive vaccine, exclamations, and openness to reach out and help others.

“*My belief is that the benefits greatly outweigh the risks of receiving this vaccine. Having had Covid this past November, I count myself among the very fortunate to be here… Every adult should have access to and receive this vaccine*.”

“*I’ve had both injections and I would do it again*.”

“*I have volunteered to be a screener, a Covid swab tester, and a vaccinator, as I feel it is my duty and call to help fight this pandemic with every tool we have. I have been encouraging everyone I know to get the vaccine through personal contact and even social media posts. I have congratulated and thanked those who receive the vaccine as I know that it will take up to 80% of all to get the vaccine to achieve ‘herd’ immunity*.”

## 4. Discussion

To date, evidence on COVID-19 vaccine acceptance has been based on intention to receive the vaccine versus actual vaccine uptake [2,3,11,12,13]. Additionally, those that focused on healthcare workers examined them as a whole [5,6,7,8,13,14,15]. To our knowledge, this is the first study to investigate the unique factors associated with actual COVID-19 vaccine uptake among nursing professionals during an active COVID-19 vaccine rollout. Among 276 nursing staff working in a large medical center, over 80% were either willing to receive or had already received the vaccine, while some remained hesitant (11.2%) or unwilling (5.2%). Those who were hesitant were more likely to report not having adequate information to make an informed decision about whether to receive the vaccine and not having adequate information about the expectations of the vaccine. Confidence in vaccine safety and greater than 10 years of work experience were associated with vaccine uptake. These findings are particularly striking, considering that the survey was conducted at a time when information regarding vaccine efficacy had become public, vaccines were available, and, as was the case in the current study, being distributed on-site, and all healthcare professionals were significantly impacted by the effect of the pandemic.

The current survey was conducted when the United States was in Phase 1A of the COVID-19 vaccine rollout, which included distribution to healthcare workers. Interestingly, we found a considerably lower rate of hesitancy and consequential higher percentage of nursing staff who intended to be vaccinated during the initial roll out, compared to previous estimates that were obtained prior to the availability of COVID-19 vaccines [5,6,7,8,16,17]. The rate of unwillingness reported in our study was similar to those described in three national surveys of healthcare workers that were conducted prior to COVID-19 vaccine availability [8,14,15]. The lower than expected vaccine hesitancy found in the current study may be attributed to an increased risk of contact with COVID-19 by the survey participants [8,18]. Biswas et al. conducted a scoping review of 35 studies examining COVID-19 vaccine hesitancy among 16,158 healthcare workers prior to the availability of the vaccine and found that having direct patient care was associated with a greater likelihood of receiving the vaccine [18]. Our study included a majority of nursing staff with direct patient contact unlike previous surveys, many of whom included hospital roles that have reduced or no contact with suspected or confirmed COVID-19 cases such as clerical staff, pharmacists, social workers, and environmental service workers. Likewise, the current study was conducted in an urban area. Healthcare workers working in non-rural areas have been found to have higher vaccine acceptance versus those who work in rural areas [18]. Change in vaccine acceptance may also vary over time as additional information about risks and promotion of safety become more widely available thus alleviating reasons for hesitancy [15]. The healthcare facility that the study was conducted in was a government organization, which had an efficient data-reporting system to provide up to date information on the vaccine to all workers. Consequently, most of the survey participants reported receiving adequate information about the COVID-19 vaccine to make an informed decision about whether to receive the vaccine and 23% of those who were initially hesitant did report that they had received the first dose of the vaccine. Those who were willing to receive the vaccine also reported confidence in the safety of the vaccine and in its effectiveness to mitigate their risk. Lack of confidence in safety and effectiveness has been shown to be determinants of COVID-19 vaccine hesitancy [5,8], suggesting this finding is likely to be of consequence.

Even with the profound impact of COVID-19 on healthcare, and with the availability of a vaccine clinic on-site, there remained a proportion of individuals with sustained hesitancy or unwillingness to receive the vaccine. The sustained vaccine hesitancy and unwillingness may be the consequence of the unknown in terms of long-term impact, which was a commonly cited reason for being unsure about accepting vaccination. One unwilling participant stated, “It is a new vaccination that was rushed, and the long-term side effects are unknown. Personally, I would rather take my chances with getting COVID than risk a vaccination.” We found hesitancy to be significantly associated with the expectation that the vaccine would not provide immunity. Unlike Fisher (2020) [2] who surveyed the general population, we found no association between vaccine hesitancy and perceived risk for COVID-19 suggesting unique attributes of nursing staff compared to the general population and the potential need for a personalized approach to vaccine campaigns.

COVID-19 vaccine uptake has not been examined among nursing staff. We found a high COVID-19 vaccine uptake rate (83.3%) among nursing staff who participated in the survey. In support of the current findings, a recent cross-sectional study addressing healthcare workers’ willingness to be vaccinated, found that out of 2761 healthcare workers across 17 healthcare institutions in Canada, 80.9% (*n* = 2233) accepted the vaccine [19]. Similar uptake rates of the seasonal influenza vaccine were found among Greek healthcare workers (flu season 2020–2021) and among Arab healthcare workers (flu season 2014–2015) [15,20]. To the contrary, one recent study among nurses in Hong Kong examining influenza uptake found only 49% self-reported influenza vaccination in the 2019–2020 season; however, 63% intended to receive the COVID-19 vaccine [17]. These results suggest that intention to receive the COVID-19 vaccine may not always follow uptake; thus, monitoring temporal changes in both concepts could provide additional benefit for future vaccine campaigns.

Our study found that confidence in vaccine safety is associated with vaccine uptake, which supports previous findings examining influenza vaccine uptake in adults and healthcare workers [21,22,23]. Interestingly, we also found that having greater than 10 years of work experience was also associated with uptake, which is a novel factor and one that should be further investigated. Evidence that these characteristics and attitudes are associated with vaccine uptake could be useful in targeting vaccine messaging and outreach to nursing staff who are at risk for not getting vaccinated. According to recent systematic reviews assessing the effectiveness of interventions to improve influenza vaccine uptake among healthcare workers, multicomponent interventions (e.g., on-site vaccination, vaccination stands with educational material, incentives, and mandates) show promise to increase vaccination within this population and may be an equally effective strategy for the COVID-19 vaccine [24,25,26].

A strength of our study is that the timing of the survey administration coincided with an active vaccine rollout making the findings particularly timely and salient regarding sustained vaccine hesitancy that continues to be evident among U.S. healthcare workers. Our study also has limitations. First, our findings may not be generalizable because it was limited to one healthcare center. Nursing staff who are men, non-White, and who work part-time with extensive work experience were likely underrepresented. Moreover, because the survey was cross-sectional in nature, we were unable to capture fluctuations in feelings over time possibly associated with the rapid exchange of information and nature of the pandemic. To ensure anonymity, participant identifiers were not collected by the web-based software platform used to administer the survey. Thus, it is possible that a participant completed the survey more than once despite being asked to disregard the survey reminder email if he/she already participated. Finally, selection bias may have existed as feelings related to the vaccine could have affected participation.

## 5. Conclusions

To our knowledge, our study is the first to examine COVID-19 vaccine uptake exclusively in nursing staff, a population that plays a significant role in combatting COVID-19. This study provides insight into nurses’ willingness and uptake of the COVID-19 vaccine and into factors associated with both concepts, which could be used to inform strategies to increase future vaccine uptake. We found that most nursing staff (over 80%) working at a large medical center who participated in the survey during an active vaccine rollout, were willing or had received the COVID-19 vaccine. Approximately 11% remained hesitant and 5% remained unwilling even with the availability of a vaccine. Confidence in vaccine safety and greater than 10 years of work experience were associated with vaccine uptake. For those who are hesitant to receive the COVID-19 vaccine, focused public health efforts to provide more data on harmful side effects and on its efficacy may help to increase the likelihood of choosing to get the vaccine. Likewise, intervention efforts may also consider partnering with professional organizations and associated scientific journals to support effective communication. Nurses are on the frontline and are facing critical staffing shortages. It is imperative to utilize data from surveys, like the current study, to implement strategies to keep this vulnerable population safe and sustain a vital healthcare workforce. In addition, the overwhelming acceptance of the COVID-19 vaccine that was observed in the current study may be used to influence other hesitant nursing staff to accept vaccination. These points are especially salient due to the emergence of new variants of SARS-CoV-2.

## Figures and Tables

**Table 1:** Participant Characteristics (*n* = 276).

Sociodemographic Variables	All ^a^	Willing ^a^	Hesitant ^a^ (Prefer to Wait)	Unwilling ^a^	*p*-Value ^b^
	*n* = 276	*n* = 226	*n* = 31	*n* = 14	
Age ^a^, mean (SD); (*n* = 275/225/31/14) (range)	48.3 (10.2) (23–69)	48.6 (10.3) (26–69)	45.5 (9.9) (23–61)	47.1 (9.3) (30–57)	0.24
Sex ^a^ (*n* = 275/226/30/14)					
Male	43 (15.6)	37 (16.4)	5 (16.1)	1 (7.1)	0.88
Female	231 (83.7)	189 (83.6)	25 (80.6)	12 (85.7)	
Other	1 (0.4)	0 (0)	0 (0)	1 (7.1)	
Race/ethnicity ^a^ (*n* = 275/225/31/14)					
White	225 (81.5)	189 (83.6)	24 (77.4)	10 (71.4)	0.68
Non-White	30 (14.5)	30 (13.3)	5 (16.1)	2 (14.3)	
Prefer not to answer	10 (3.6)	6(2.7)	2 (6.5)	2 (14.3)	
Marital Status					
Married/Partnered	178 (64.5)	148 (65.5)	18 (58.1)	9 (64.3)	0.59
Single	91 (33.0)	73 (32.3)	13 (41.9)	4 (28.6)	
Prefer not to answer	7 (2.5)	5 (2.2)	0	1 (0.4)	
Employment Status ^a^ (*n* = 275/226/30/14)					
Full-time	262 (94.9)	215 (95.1)	29 (93.5)	13 (92.9)	0.69
Part-time	13 (4.7)	73 (32.3)	1 (3.2)	1 (7.1)	
Work Experience					
<= 10 years	58 (21.0)	42 (18.6)	12 (38.7)	4 (28.6)	0.03 *
>10 years	218 (79.0)	184 (81.4)	19 (61.4)	10 (71.4)	
Type of Nursing Staff					
Registered Nurse	189 (68.5)	152 (67.3)	27 (87.1)	8 (57.1)	0.43
Licensed Practical Nurse	25 (9.1)	18 (8.0)	3 (9.7)	3 (21.4)	
Nurse Practitioner	30 (10.9)	26 (11.5)	1 (3.2)	1 (7.1)	
Clinical Nurse Specialist or Educator	5 (1.8)	5 (2.2)	0	0	
Nursing Assistant	17 (6.2)	15 (6.6)	0	2 (14.3)	
Administrator	6 (2.2)	6 (2.7)	0	0	
Other	4 (1.4)	4 (1.8)	0	0	

Unless otherwise indicated, data are presented as number (percentage) of survey respondents. * *p*-value < 0.05. ^a^ No. of participants unless otherwise noted (where numbers are shown for total sample/willing/hesitant/unwilling). ^b^ Denotes *p* values from χ^2^ tests (Fisher’s exact test if less than five cases were present in a cell) for categorical variables, *t* tests for continuous variables.

**Table 2:** COVID-19 related responses by vaccine hesitancy.

Vaccine-Related Variables	All ^a^	Willing ^a^	Hesitant ^a^	Unwilling ^a^	*p* Value ^b^
	(*n* = 276)	*n* = 226	*n* = 31	*n* = 14	
What is your best guess as to whether you will get COVID-19? ^a^ (*n* = 273/223/31/14)					
I don’t think I will get it	92 (33.3)	82 (36.3)	6 (19.4)	4 (28.6)	0.17
I think I will get a mild case	56 (20.3)	41 (18.4)	9 (29.0)	6 (42.9)	
I think I will get seriously ill	19 (6.9)	16 (7.1)	3 (9.7)	0	
I have already had it	106 (38.4)	84 (37.2)	13 (41.9)	4 (28.6)	
Did you have adequate information about the expectations of the vaccine? ^a^ (*n* = 270/222/31/14)					
Yes	252 (91.3)	215 (96.8)	21 (67.7)	13 (92.9)	<0.001 *
No	18 (6.5)	7 (3.2)	10 (32.3)	1 (7.1)	
Did you have adequate information to make an informed decision about whether to receive the vaccine or not? ^a^ (*n* = 272/225/31/11)					
Yes	249 (90.2)	216 (96.0)	17 (54.8)	11 (100.0)	<0.001 *
No	23 (8.3)	9 (4.0)	14 (45.2)	0	
How confident are you in the safety of the vaccine? ^a^ (*n* = 272/224/30/13)					
Completely/fairly	204 (73.9)	192 (85.7)	7 (23.3)	0	<0.001 *
Somewhat	35 (12.7)	23 (10.3)	10 (33.3)	2 (15.4)	
Slightly/not at all	33 (12.0)	9 (4.0)	13 (43.3)	11 (84.6)	
How confident are you in the effectiveness of the vaccine? ^a^ (*n* = 270/226/31/13)					
Completely/fairly	199 (72.1)	183 (81.0)	11 (35.5)	1 (7.7)	<0.001 *
Somewhat/slightly/not at all	76 (27.5)	43 (19.0)	20 (64.5)	12 (92.3)	
What are your expectations of the effectiveness? ^a^ (*n* = 274/225/31/13)					
Limited/Lifetime Immunity	199 (72.6)	175 (77.8)	16 (51.6)	4 (30.8)	<0.001 *
Reduction in symptom severity only/completely ineffective	75 (27.4)	50 (22.2)	15(48.4)	9 (69.2)	
How knowledgeable are you about the development process of the vaccine? ^a^ (*n* = 274/225/31/13)					
A great deal/fairly/somewhat	235 (85.1)	204 (90.7)	17 (54.8)	10 (76.9)	<0.001 *
A little/not at all	39 (14.1)	21 (9.3)	14 (45.2)	3 (23.1)	

Unless otherwise indicated, data are presented as number (percentage) of survey respondents. Comparisons are made between willing, hesitant, and unwilling groups only. * *p*-value < 0.05. ^a^ No. of participants unless otherwise noted (where numbers are shown for total sample/willing/hesitant/unwilling). ^b^ Denotes *p* values from χ^2^ tests (Fisher’s exact test if less than five cases were present in a cell) for categorical variables, *t* tests for continuous variables.

**Table 3:** Sociodemographic and COVID-19 Vaccine Variables by Vaccine Uptake.

Sociodemographic Variables	Received	Did Not Receive	*p*-Value ^b^
	*n* = 230	*n* = 40	
Age, mean (SD) (range)	48.31 (10.5) (23–69)	47.3 (8.7) (34–61)	0.58
Sex ^a^ (*n* = 230/39)			
Male	39 (17.0)	4 (10.0)	0.32
Female	191 (83.0)	34 (85.0)	
Other	0	1 (2.6)	
Race/ethnicity ^a^ (*n* = 229/40)			
White	191 (83.0)	31 (77.5)	0.72
Non-White	31 (13.5)	6 (15.0)	
Prefer not to answer	7 (3.1)	3 (7.5)	
Marital Status			
Married/Partnered	150 (65.2)	24 (60.0)	0.53
Single	75 (32.6)	15 (37.5)	
Prefer not to answer	5(2.2)	1(2.5)	
Employment Status ^a^ (*n* = 230/39)			
Full-time	220 (95.7)	36 (90.0)	0.37
Part-time	10 (4.3)	3 (7.5)	
Work Experience 2 Groups			
<=10 years	44 (19.1)	14 (35.0)	0.024 *
>10 years	186 (80.9)	26 (65.0)	
What is your best guess as to whether you will get COVID-19? ^a^ (*n* = 227/40)			
I don’t think I will get it	83 (36.6)	9 (22.5)	0.28
I think I will get a mild case	44 (19.4)	12 (30.0)	
I think I will get seriously ill	15 9 (6.6)	3 (7.5)	
I have already had it	85 (37.4)	16 (40.0)	
Did you have adequate information about the expectations of the vaccine? ^a^ (*n* = 226/40)			
Yes	218 (96.5)	30 (75.0)	<0.001 *
No	8 (3.5)	10 (25.0)	
Did you have adequate information to make an informed decision about whether to receive the vaccine or not? ^a^ (*n* = 229/37)			
Yes	218 (95.2)	25 (67.6))	<0.001 *
No	11 (4.8)	12 (32.4)	
How confident are you in the safety of the vaccine? ^a^ (*n* = 227/39)			
Completely/fairly	192 (84.6)	6 (15.4%)	<0.001 *
Somewhat	24 (10.6)	11 (28.2)	
Slightly/not at all	11 (4.8)	22 (56.4)	
How confident are you in the effectiveness of the vaccine? ^a^ (*n* = 230/39)			
Completely/fairly/	183 (79.6)	11 (28.2)	<0.001 *
Somewhat/slightly/not at all	47 (20.4)	28 (71.8)	
What are your expectations of the effectiveness? ^a^ (*n* = 229/225/39)			
Lifetime/Limited Immunity	178 (77.7)	17 (43.6)	<0.001 *
Reduction in symptom severity only/completely ineffective	51 (22.3)	22 (56.4)	
How knowledgeable are you about the development process of the vaccine? ^a^ (*n* = 229/39)			
A great deal/fairly/somewhat	208 (90.8)	22 (56.4)	<0.001
A little/not at all	21 (9.2)	17 (43.6)	

Unless otherwise indicated, data are presented as number (percentage) of survey respondents. Comparisons are made between those who received the vaccine and those who did not receive the vaccine. * *p*-value < 0.05. ^a^ No. of participants unless otherwise noted (where numbers are shown for received vaccine/did not receive vaccine). ^b^ Denotes *p* values from χ^2^ tests (Fisher’s exact test if less than five cases were present in a cell) for categorical variables, *t* tests for continuous variables.

**Table 4:** Multivariate associations with COVID-19 vaccine uptake among nursing staff at a large medical center (*n* = 276).

	Predictor	Vaccinated vs. Not Vaccinated		
		Odds Ratio	95% CI	
			Lower Bound	Upper Bound
Step 1	^a^ Confidence in Safety	7.48	4.41	12.69
Step 2	^b^ >10 years. Work Experience	3.05	1.16	8.00
	^b^ Confidence in Safety	7.78	4.49	13.46
	^c^ Adequate info about Expectations of Vaccine			
	^c^ Adequate info to make informed decision			
	^c^ Confidence in Effectiveness			
	^c^ Expectations of Effectiveness			

Note: Binary Dependent variable = vaccine receipt; forward selection method used. ^a^ Variable entered on step 1: confidence in safety. ^b^ Variables entered in step 2: Work Experience. ^c^ Variables not entered into the model.

## Data Availability

The data set used and/or analyzed during the present study are available from the corresponding author on reasonable request.
